# Detection of Neuromuscular Deficits in Movement Pattern among Uninjured Federated Youth Basketball Players: A Cross-Sectional Study

**DOI:** 10.3390/ijerph19074077

**Published:** 2022-03-29

**Authors:** Cristina Adillón, Montse Gallegos, Silvia Treviño, Isabel Salvat

**Affiliations:** 1 Department of Medicine and Surgery, Faculty of Medicine and Health Sciences, Institut d’Investigació Sanitària Pere Virgili, Universitat Rovira i Virgili, 43204 Reus, Spain; mariaisabel.salvat@urv.cat; 2 Health Department, Catalan Basketball Federation, 08018 Barcelona, Spain; mgallegosma@gmail.com (M.G.); doctoratrevi@gmail.com (S.T.)

**Keywords:** adolescent, pediatrics, postural balance, range of motion, primary prevention, basketball

## Abstract

(1) Background: The aim of the present study was to evaluate and to detect neuromuscular deficiencies in static and dynamic tests among federated youth basketball players. (2) Methods: Cross-sectional study with 778 basketball players. Specific tests and trials were conducted to evaluate members of teams from several clubs in male and female from under 12 (U12) to under 17 (U17) categories. The evaluations consisted of static physical measurements and dynamic measurements. (3) Results: 575 players were included in this study. A total of 95% of participants are unable to keep their ankle stable in monopodial loading; 86% present dynamic lower extremity valgus with statistically significant differences between categories (*p* = 0.004); 94% are unable to keep the pelvis stable when performing a single-leg squat; 93% are unable to keep their trunk stable when performing the same movement. During landing, 96% present dynamic lower extremity valgus. The thighs of 92% do not reach parallel (peak of jump). (4) Conclusions: The most frequent neuromuscular deficits in federated youth basketball players are related to instability, the most frequent being ankle instability, followed by lumbo-pelvic instability, dynamic postural instability and dynamic knee valgus. Deficits in jumping/landing technique are also very frequent in all the items analyzed (jumping, landing and plyometrics). The performed tests, which mostly showed a poor performance by the sample, can be indicative of injury probability.

## 1. Introduction

Basketball has become an increasingly physical game in which contact is accepted and expected [1]. It is considered a high average injury incidence sport, with 10 injuries per 1000 h of exposure [2,3]. Unlike other team sports, it combines a high proportion of high intensity and explosive actions, including running, turning, accelerating, jumping and landing, interspersed with frequent changes of direction, decelerating and stops [4]. These actions are very demanding on the lower limbs and increase the risk of lower extremity injury [5]. The most frequent injuries occur during training (67%), especially in the ankle (30–48%) and the knee (18%) [6,7,8,9,10]. Although males are more often affected by such injuries, in females, they lead to a longer recovery time and inability to compete [10,11]. The most vulnerable group is adolescents [12,13]. These players may not be in the same physical condition as those playing at higher levels. An excessive and overly rapid increase in the training load may cause negative adaptations, which in turn can lead to injury and illness [14,15].

In contrast, almost half of all injuries are estimated to be caused by a non-contact mechanism [16], which makes them preventable. Therefore, to reduce and minimize the incidence of injury, research studies should aim to identify players’ intrinsic risk factors, extrinsic risk factors and triggering events, and, from that point of departure, try to correct or compensate for modifiable factors [17,18,19]. Screening for these modifiable risk factors can help define high-risk populations, since the more risk factors a player has, the more likely he or she is to be injured [11].

The most common situations in which ankle and knee injuries occur in basketball are in defending actions, rebounding capture (18–29%) and losing balls (24%) [7,8]. These actions involve jumping and landing, whether to take a stride or to run or catch the ball [20]. From a biomechanical perspective, landings require optimal technical performance to ensure efficient absorption of the impact forces generated by foot–floor contact and thus minimize the risk of injury. Landing has been associated with ankle sprains, patellar tendon injuries and anterior cruciate ligament ruptures [20].

In order to perform these actions with the least possible risk, players must develop specific motor skills: shooting, rebounding, passing and dribbling, blocking, stealing and, again, rebounding [21,22,23,24]. However, it should be noted that younger basketball players may have more difficulties in performing movements specific to basketball, since they are in the process of developing basic motor skills [25,26] related to coordination and balance, which are fundamental in the ability to develop more complex motor learning for the different technical-tactical demands of basketball [23,24].

In addition, it should be considered that joints, muscles and tendons do not always grow synchronously [19]. This consideration, combined with poorly coordinated growth, may give rise to the adoption of poor movement patterns in sports where jumping is the primary action. Such patterns can lead to imbalances in mobility and stability, which have been identified as risk factors for injury [18,19].

Most of the current published literature about ankle and knee injuries focuses on the circumstances surrounding the injury. However, evidence regarding the risk factors associated with them is limited [22]. Most of the literature on sports injuries among children and adolescents is based on descriptive data on the magnitude of the injury. These studies generally focus on individual sports and include studies of adults [6].

A global and comprehensive review of injury risk factors in child and adolescent sport is needed to provide guidance for future research on injury prevention in this population [13].

Consequently, the main aim of this study is to evaluate and identify neuromuscular deficits among federated youth basketball players (under 12 to under 17) in mobility, stability and landing technique in similar static and dynamic tests that simulate the most common actions required in basketball.

## 2. Materials and Methods

### 2.1. Study Design

This cross-sectional study was conducted from October 2018 to May 2019. The study adhered to the tenets of the Declaration of Helsinki and received ethical approval from the local institutional review board (Pere Virgili Institute; Ref. CEICm: 123/2018). The study protocol was registered with ClinicalTrials.gov ID: NCT04796753.

Informed consent for participation in the study was obtained from the children and their parents or guardians.

### 2.2. Participants

The participants were basketball players who belonged to youth basketball developmental teams in the under 12 (U12), under 14 (U14), under 16 (U16) and under 17 (U17) categories. All participants were recruited by means of simple random sampling from the Catalan Basketball Federation during the 2018–2019 season. The participants were classified according to gender and age as stipulated by the rules determined annually for the respective official competitions. The study was carried out in the facilities of each club. The inclusion criteria were being age ≥12 and <18 at testing and actively competing during the study. Subjects were excluded if they had sustained any type of injury in the lower limbs before screening; presented any injury (overuse or acute) at the time of testing; if they had any oncological, psychological and/or psychiatric illnesses; or if they did not attend on the day of the assessment. The final sample size was a convenience sample, determined by the number of players who agreed to participate voluntarily.

### 2.3. Outcomes

Primary outcome measures: ankle joint dorsiflexion was evaluated with the weight-bearing lunge test through the LegMotion system [27,28]; monopodial ankle stability was analyzed using the single-leg balance test [29]; dynamic lower extremity valgus was analyzed with the single-leg squat test [30,31,32,33]; lumbopelvic stability and dynamic postural control were assessed by means of the single-leg squat test, hurdle step test and modified tuck jump test [34,35]; and neuromuscular deficits during continuous tuck jumps, landing technique flaws and plyometric technique were evaluated with the modified tuck jump test [36,37]. All of these tests were validated for these purposes. All players were familiarized beforehand with the procedures of all tests. Testing was performed on the same day, in the same order and at the same time of day (6.00 to 8.00 p.m.).

Secondary outcome measures: age, age categories, gender, weight, height, wingspan and body mass index were recorded. The presence of hypermobility was evaluated by Beighton’s criteria (scores of ≥7 points out of a total of 9 points were considered hypermobile) [38], and the presence of lower limb dominance was observed with the criteria described by Harris on foot dominance [39].

### 2.4. Procedure

All participants completed the same 10 min neuromuscular warm-up consisting of the following exercises: joint mobility exercises, dynamic stretching exercises, jumps, multidirectional displacements and changes of direction. Afterward, subjects were allowed three practice trials for each test in this order: the single-leg balance test, the weight-bearing lunge test, the hurdle step test, the single-leg squat test and the modified tuck jump test. Consistent feedback was provided throughout to ensure proper technique. The performance of each test was recorded using two cameras (IPhone XS, Apple, Cupertino, CA, USA). In order to allow visible tracking of the different joints, participants were required to wear shorts with the hem at approximately mid-thigh. When scoring performance, each test was viewed in sagittal and frontal views. The raters watched the videos as many times as necessary to score each test. For a more specific analysis, drawing tools were used to take measurements included in the application Hudl Technique version 6.0.0 (Agile Sports Technologies, Inc., Lincoln, NE, USA). Researcher 1 was in charge of scoring the following tests: the hurdle step test, the single-leg squat test and the modified tuck jump test. Researcher 2 was assigned the single-leg balance test and the weight-bearing dorsiflexion test. Researcher 3 was responsible for recording the secondary variables. All raters had over five years of clinical experience and previous training and experience with scoring from video replay.

### 2.5. Data Sources/Measurement

The valuation procedures used to obtain the value of each of the variables are explained below:

**Single-leg balance test:** A stopwatch was used to evaluate whether the player was able to maintain the monopodal position with eyes closed for 10 s or whether the player lost balance during that time. It was considered stable if the foot remained in neutral position during the movement and unstable if excessive pronation of the foot during the movement or external rotation of the leg was evident [29].

**Weight-bearing lunge test:** Ankle dorsiflexion was evaluated through the LegMotion system (LegMotion, your MOtion^®^, Albacete, Spain). Subjects were instructed to try to bring the knee to touch the metal rod (initially placed at a distance of 10 cm) without lifting the heel off the ground. The distance achieved was recorded in centimeters [27,28].

**Hurdle step test:** It consists of overcoming an obstacle located below the knee by means of triple flexion of the limb to be assessed. For this purpose, the Leg Motion^®^ system (LegMotion, your MOtion^®^, Albacete, Spain) was used, and the rope was adjusted to the height of the anterior tuberosities of the participant’s tibiae. Lumbopelvic stability was considered if there was minimal movement in all three planes, the pelvic girdle was aligned, and there was no evidence of excessive anteroposterior tilt and/or trunk rotation. It was considered unstable if these criteria were not met [34,35].

**Single-leg squat test:** It consists of performing a monopodal squat to test dynamic knee valgus and dynamic postural control. They were to perform a single-leg squat to 30º knee flexion. The knee was considered to be aligned if the patella was over the second toe. On the other hand, it was considered dynamic postural control if there was minimal translation of the center of mass, i.e., no lateral flexion/tilt, rotation or trunk flexion/extension [30,31,32,33].

**Modified tuck jump test:** This test consists of performing continuous jumps of maximum height with the knees to the chest inside a rectangle of 41 × 35 cm during 10 s. The results were analyzed qualitatively according to the ten criteria described by Fort-Vanmeerhaeghe [36,37].

### 2.6. Bias

To minimize observation bias, the researcher in charge of analyzing the results did not know the hypothesis of the study and used measuring instruments with previously established evaluation criteria.

### 2.7. Statistical Analyses

Statistical analyses were performed using SPSS version 26.0 (Statistical Package for the Social Sciences for Windows; IBM Corp, Armonk, NY, USA). The normality of each variable was confirmed by means of the Shapiro–Wilk test. Normally distributed data for continuous variables were summarized with means and standard deviations (SD). Qualitative variables were described as absolute frequencies and percentages. To compare neuromuscular deficits between genders, the chi square test and independent-sample Student’s *t*-test were used. Meanwhile, a one-way ANOVA test was used to examine mean differences among different categories. For all tests, *p*-values were two sided. A *p*-value less than 0.05 was considered significant.

## 3. Results

### 3.1. Description of Sample

**Figure 1:**
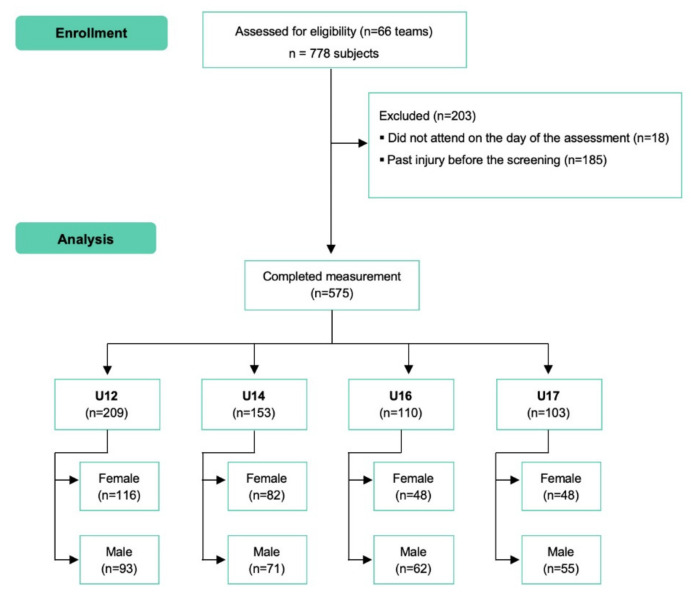
Flow diagram.

A total of 185 players were excluded from the study because of past injury prior to screening and 18 did not attend on the day of the assessment (Figure 1). In the end, 575 youth basketball players who met the eligibility criteria and volunteered to participate were included in the study. The mean (SD) age is 13.15 (2.15), and 51% of the participants are female.

The descriptive characteristics for anthropometric data are reported in Table 1. All data were found to be normally distributed. As would be expected based on maturity, U17 players were taller, heavier and had a larger wingspan compared to the other players. In all categories, 10% of the players presented generalized hyperlaxity, and most of them were right handed.

### 3.2. Ankle Joint Dorsiflexion

The mean for ankle joint dorsiflexion (SD) was 10.50 (2.45) cm, with no statistically significant differences between genders or between dominant leg/non-dominant leg. However, there were statistically significant differences between age categories (*p* < 0.001). The U12 category exhibited more limited mobility than the higher age categories (see Table 2).

### 3.3. Functional Ankle Stability

The analysis of ankle stability showed that 94.90% of the players were unable to remain stable during monopodial loading, with no statistically significant differences between categories (*p* = 0.877). U16 players exhibited the highest levels of ankle stability, especially girls in the non-dominant extremity. None of these differences were found to be statistically significant between genders (Table 3).

### 3.4. Dynamic Lower Extremity Valgus

Eighty-six percent of the players presented a dynamic lower extremity valgus (Table 4) with statistically significant differences between categories (*p* = 0.004). U12 had the highest percentage of dynamic valgus and U16 the lowest. The other two categories yielded similar values, with U17 boys showing statistically significant differences also between the dominant and non-dominant limb (*p* = 0.044). No statistically significant differences were found between the genders (*p* = 0.101).

### 3.5. Lumbopelvic Stability and Dynamic Postural Control

Six percent of participants were able to perform a single-leg squat while maintaining a stable pelvis, with no difference between the genders (*p* = 0.388), except in the U12 category (Table 5). The analysis by categories showed statistically significant differences between them (*p* < 0.001) with stability percentages below 5% in the U12, U14 and U17 categories. In the U16 male category, in contrast, 19% of players were able to maintain their pelvis stable when performing a single-leg squat.

Likewise, males had greater control (9%) than females (5%), although gender was found to be statistically significant (*p* = 0.047). The difference was only statistically significant in the U17 category (see Table 5). Analyzing the results by category revealed statistically significant differences in males (*p* < 0.001) with an age-related progression from 2% in U12 to 15% in U17. However, this increase in dynamic postural control was not observed in the female categories (*p* = 0.360).

### 3.6. Neuromuscular Deficits during Continuous Tuck Jumps

The values for items assessing jumping/landing technique showed that approximately 4% of the players did not present valgus in the lower extremity during landing. In addition, 8% were able to maintain the knees higher or at the same level as the hips in the peak of the jump, and 3% were able to maintain the thighs equal side to side during flight. No statistically significant differences were found based on gender (see Table 6).

Analyzing the position of the foot during landing, 6% of players were observed to have foot placement exactly shoulder width apart. Statistically significant differences were found between genders only in the U17 category. Similarly, 4% of the subjects exhibited parallel foot placement (at the toes), 7% exhibited equal side-to-side foot contact timing, and 8% exhibited subtle noise at landing (landing on the balls of their feet) (Table 6).

A total of 9% of the subjects were able to perform reactive and reflex jumps, 4% showed no decline in technique, and 5% landed in the same footprint, with no statistically significant differences found based on gender or category in any of the items assessed (Table 6).

## 4. Discussion

Federated youth basketball players (U12 to U17) presented high percentages of neuromuscular deficits in mobility, stability and jumping/landing technique. These findings indicate that there are many and diverse inadequate movement patterns and neuromuscular deficiencies in the simulation of all the most used actions in basketball in all age categories, without great variation between genders, categories or dominant and non-dominant limbs. Several authors have related these deficiencies to the most common athletic musculoskeletal injuries [10,40,41].

The values for dorsal ankle flexion under load were similar to those described for the adult population, with the U12 category presenting lower values. These values coincide with the results described by Gonzalo-Skok et al. [42] in a similar study, especially among U14 basketball players. Although mobility exercise that focuses on increasing ankle joint dorsiflexion is currently one of the most prevalent exercises in injury prevention programs, consideration should be given to whether this population needs to increase this range of motion and whether this can be considered a risk factor for injury in uninjured youth basketball players.

For ankle stability, 95% of players were unable to maintain monopodial stability with no differences related to gender or category. No differences were found between the dominant and non-dominant limb, which was consistent with other studies [43]. Halabchi et al. [44] state that elite basketball and football players (age 15 to 40 years) who have sustained an ankle sprain present a deficit in ankle stability and impaired single-leg balance test. This relationship should be revised based on the results of the present study conducted with players who had never had an ankle injury and whose instability rates were higher than those found by those authors. Consideration should be given to whether these deficits are a cause or a consequence of injury, an aspect to be taken into account during training, and, above all, in the physical conditioning of these players.

Eighty-six percent of the players presented a dynamic lower extremity valgus, with the U12, U14 and U17 categories presenting the highest percentages. Boys under 16 were the best performers. However, this should be investigated to determine why these results worsen in boys under 17; it may be due to the hours dedicated to physical conditioning or the exercises they perform in these sessions. These results are consistent with those obtained by Agresta et al. [13], who found that younger children had poorer performance, suggesting that balance may still be developing. However, although limb dominance has been indicated as having an influence on uneven lower limb strength [45], no differences were observed in the present study between the dominant and non-dominant limb, except in boys under 17. Furthermore, males presented a higher percentage of knee valgus than females, although the difference was not statistically significant.

About 94% of the players presented lumbopelvic instability, a percentage that decreased to 81% in boys under 16. Weakness of the hip abductors predisposes players to increased adduction and internal rotation of the hip, which in turn leads to increased medial motion and knee valgus [46]. This factor could be related to the improvement of dynamic knee valgus. This is more pronounced when the player performs a single-leg squat and on landing after a jump. This has been shown to have implications for anterior cruciate ligament injury [19,47].

In terms of lack of dynamic postural control, 94% of the players were unable to maintain a stable trunk while performing a single-leg squat. The progression in the improvement of control from the U12 to U17 categories is noteworthy, with better overall dynamic postural control exhibited by males. In the U17 male category, the percentage dropped to 82.20%, with a statistically significant difference between the U17 female and U17 male categories. These results show deficient dynamic postural control, which is relevant in relation to anterior cruciate ligament injuries, since current evidence shows that a stable trunk decreases the stress on this ligament in a single-leg squat by 24% [48,49]. Dynamic postural control and neuromuscular disturbances are also considered intrinsic risk factors for ankle sprain [50] and anterior cruciate ligament injury [49], especially poor strength and related instability of the lumbopelvic region [48,51].

The jumping analysis showed that more than 90% of players were unable to perform a jump with the optimal technique to ensure efficient absorption of the impact forces of foot–floor contact. This is relevant in a sport such as basketball, in which jumping technique plays an important role in improving vertical jump height, which influences shooting, rebounding and shot-blocking skills. In fact, the results of the present study are in agreement with those of Cowling and Steele [52], who found better patterns of muscle synchrony in men than in women. The results presented, in accordance with the criteria described by Fort-Vanmeerhaeghe et al. [36], do not specify whether the landing is rigid or if there is an excess of range of motion. This is an item that could be added to better assess the lateral evaluation view of the modified tuck jump assessment, since if a player has a limited range of motion during shock absorption on landing after a jump, especially in the ankle, knee and hip joints, it could lead to bone injury. However, an excessive range of motion in the joints could cause musculoskeletal injuries [53]. Therefore, it is also important to analyze whether players actively flex the ankles, knees and hips during the impact absorption phase of foot–floor contact to dampen reaction forces, as several studies have confirmed that active triple flexion of the lower limbs is the key factor for impact force attenuation and energy absorption, thus decreasing the risk of ligament injury on landing [20,54,55]. It may be due to the specificity of training and the effect of the specific load of basketball, which will consequently create deficits and may jeopardize the health and physical integrity of athletes.

The results of the present study reveal that most of the participants employ an inappropriate landing technique, which, in conjunction with the musculoskeletal imbalances identified, may predispose them to injury [8,56]. However, the limitations of the modified tuck jump assessment prevent us from inferring which types of injuries the players analyzed are more predisposed to.

While the mobility values identified may be considered normal, very high percentages of players presented alterations in stability and jumping/landing technique. The scarcity of epidemiological studies with similar analyses of youth basketball players (U12 to U18) prevents us from comparing our results with those of other publications.

The values found in this study may offer a basis for establishing reference values, since there are currently no reference values in the pediatric and juvenile population, despite this group having been described as the most vulnerable to injury [12].

Functional deficiencies are easy to assess with accessible, cost-effective and easy-to-perform tests in clinical practice, which could be incorporated into a test battery to decrease the susceptibility of basketball players to injury. Identifying the risk of injury among athletes and implementing appropriate protocols can help coaches, basketball athletic trainers and health specialists to reduce the incidence of injury and improve training methods. These factors have often been studied separately when the risk of injury is multifactorial. In youth basketball players, ankle and knee injuries are commonplace and can result in exclusion from competition, often for long periods of time [26].

Some limitations can be found in this study. Injuries are multifactorial, and despite having identified alterations in movement patterns as possible modifiable injury risk factors, emotional and psychosocial factors, which are also related to increased incidence of injuries in this sport, have yet to be analyzed. Currently, there are no reference values among children for loaded ankle dorsiflexion and other variables. Further studies are needed to establish such benchmarks. Likewise, although neuromuscular deficits can be measured, objectively quantified and compared with a standard pattern, or with some validated or calculated reference values, specific qualitative scoring criteria should be established to detect alterations in movement patterns. The findings in this study are exploratory and results should be considered cautiously as a type I error may have been incurred.

In the future, it should also be noted that fatigue (physiological, neurological and psychological) affects physical performance, changes the efficiency of contractile capacity in extrafusal muscle fibers and challenges the efficiency of afferent information from muscle strands, which ultimately alters neuromuscular control and negatively affects dynamic postural control. In addition, it would be interesting to analyze whether an exercise program designed to correct alterations in movement patterns decreases modifiable risk factors and player susceptibility, and, in the long term, reduces the incidence of injuries in youth basketball players.

## 5. Conclusions

In summary, the most frequent neuromuscular deficits in federated youth basketball players are related to instability, the most frequent being ankle instability, followed by lumbo-pelvic instability, dynamic postural instability and dynamic knee valgus. Deficits in jumping/landing technique are also very frequent in all the items analyzed (jumping, landing and plyometrics). The performed tests, which mostly showed a poor performance by the sample, can be indicative of injury probability. Identifying and correcting these intrinsic functional deficits can be the key for implementing exercise programs in order to reduce the incidence of injury.

## Figures and Tables

**Table 1:** Anthropometric data for between-group comparisons of U12 to U17 basketball players.

Outcomes	U12 (*n* = 209)	U14 (*n* = 153)	U16 (*n* = 110)	U17 (*n* = 103)
Gender ^a^, female	116 (56.00%)	82 (54.00%)	48 (44.00%)	48 (47.00%)
Weight ^b^, kg	44.04 (8.08)	53.71 (10.76)	63.76 (13.50)	67.27 (12.89)
Height ^b^, cm	154.70 (7.45)	163.89 (8.92)	173.30 (14.59)	176.94 (11.75)
Wingspan ^b^, cm	152.97 (11.26)	164.07 (14.45)	174.48 (11.27)	178.77 (13.98)
BMI ^b^, kg/m^2^	18.31 (2.48)	19.89 (3.00)	21.11 (3.56)	21.42 (3.06)
Hypermobility ^a^	10 (5.00%)	14 (9.00%)	14 (13.00%)	12 (12.00%)
Right handed ^a^	182 (87.00%)	139 (91.00%)	101 (92.00%)	73 (89.00%)

Abbreviations: BMI, body mass index; m, meter; kg, kilogram, cm, centimeter. Data are reported as ^a^ n (%) or ^b^ as mean (standard deviation) % (percentage).

**Table 2:** Assessment of ankle joint dorsiflexion for between-group comparisons of U12 to U17 basketball players.

Gender	Age Categories	Dominant Leg			Non-Dominant Leg			*p* Value
		Mean (SD)	SE	Interval	Mean (SD)	SE	Interval	
Female	U12 (*n* = 116)	10.54 (2.42)	0.25	[4.00, 19.50]	10.26 (2.55)	0.23	[2.00, 16.00]	0.371
	U14 (*n* = 82)	11.05 (2.49)	0.32	[6.00, 17.00]	11.36 (2.68)	0.34	[6.00, 17.00]	0.500
	U16 (*n* = 48)	11.32 (1.61)	0.26	[7.50, 14.50]	11.01 (1.65)	0.27	[6.50, 15.00]	0.859
	U17 (*n* = 48)	11.03 (2.05)	0.34	[8.00, 16.50]	11.23 (2.07)	0.34	[8.00, 16.00]	0.674
Male	U12 (*n* = 93)	9.29 (2.26)	0.25	[4.00, 17.00]	9.57 (2.08)	0.23	[4.50, 17.00]	0.399
	U14 (*n* = 71)	10.52 (2.61)	0.37	[4.50, 16.00]	10.60 (2.45)	0.34	[5.00, 17.00]	0.876
	U16 (*n* = 62)	11.27 (2.95)	0.37	[3.00, 19.00]	11.46 (2.89)	0.36	[2.50, 19.00]	0.877
	U17 (*n* = 55)	10.21 (1.85)	0.28	[6.50, 15.50]	10.07 (2.13)	0.32	[5.00, 15.50]	0.732

Abbreviations: SD, standard deviation; SE, standard error. Values are centimeters of ankle joint dorsiflexion. *p* values were obtained by independent-sample Student’s *t*-test.

**Table 3:** Ankle instability for between-group comparisons of U12 to U17 basketball players.

Gender	Age Categories	Dominant Leg	Non-Dominant Leg	*p* Value
Female	U12 (*n* = 116)	111 (96.10%)	113 (97.80%)	0.472
	U14 (*n* = 82)	79 (96.00%)	77 (93.70%)	0.315
	U16 (*n* = 48)	45 (94.70%)	43 (89.50%)	0.395
	U17 (*n* = 48)	48 (100%)	47 (97.30%)	0.314
Male	U12 (*n* = 93)	89 (95.70%)	86 (93.00%)	0.349
	U14 (*n* = 71)	70 (98.40%)	71 (100%)	0.539
	U16 (*n* = 62)	61 (98.40%)	60 (96.90%)	0.512
	U17 (*n* = 55)	51 (93.30%)	86 (93.00%)	1.000

Data are reported as *n* (%). *p* values were obtained by chi square test.

**Table 4:** Dynamic lower extremity valgus for between-group comparisons of U12 to U17 basketball players.

Gender	Age Categories	Dominant Leg	Non-Dominant Leg	*p* Value
Female	U12 (*n* = 116)	105 (82.70%)	113 (89.00%)	0.150
	U14 (*n* = 82)	49 (79.00%)	53 (85.50%)	0.347
	U16 (*n* = 48)	40 (84.20%)	40 (84.20%)	1.000
	U17 (*n* = 48)	39 (81.10%)	40 (83.80%)	0.760
Male	U12 (*n* = 93)	79 (95.20%)	80 (96.40%)	0.699
	U14 (*n* = 71)	47 (92.20%)	44 (86.30%)	0.338
	U16 (*n* = 62)	47 (76.60%)	47 (75.00%)	0.798
	U17 (*n* = 55)	45 (82.20%)	53 (95.60%)	0.044 *

Data are reported as n (%). *p* values were obtained by chi square test. * statistically significant (*p* < 0.05).

**Table 5:** Lumbopelvic and postural instability for between-group comparisons of U12 to U17 basketball players.

Outcome	Age Categories	Female (*n* = 294)	Male (*n* = 281)	*p* Value
Lumbopelvic instability	U12 (*n* = 209)	110 (94.50%)	93 (100%)	<0.001 **
	U14 (*n* = 153)	81 (98.40%)	67(94.10%)	0.728
	U16 (*n* = 110)	47 (97.40%)	50 (81.30%)	0.007 *
	U17 (*n* = 103)	48 (100%)	54 (97.80%)	0.197
Dynamic postural control deficiencies	U12 (*n* = 209)	111 (96.10%)	93 (100%)	0.067
	U14 (*n* = 153)	79 (96.80%)	65 (92.20%)	0.276
	U16 (*n* = 110)	43 (89.50%)	53 (85.90%)	0.605
	U17 (*n* = 103)	45 (94.60%)	43 (77.80%)	0.032 *

Data are reported as *n* (%). *p* values were obtained by chi square test. * statistically significant (*p* < 0.05); ** statistically significant (*p* < 0.001).

**Table 6:** Modified tuck jump assessment for between-group comparisons of U12 to U17 basketball players.

Item	Age Categories	Female (*n* = 294)	Male (*n* = 281)	*p* Value
Lower extremity valgus at landing	U12 (*n* = 209)	111 (96.10%)	91 (97.60%)	0.547
	U14 (*n* = 153)	77 (93.50%)	65 (92.20%)	0.774
	U16 (*n* = 110)	44 (92.10%)	51 (82.80%)	0.187
	U17 (*n* = 103)	44 (91.90%)	45 (82.20%)	0.201
Thighs do not reach parallel (peak of jump)	U12 (*n* = 209)	94 (81.10%)	75 (80.70%)	0.945
	U14 (*n* = 153)	71 (87.10%)	64 (90.20%)	0.607
	U16 (*n* = 110)	47 (97.40%)	47 (76.60%)	0.005 *
	U17 (*n* = 103)	39 (81.10%)	45 (82.20%)	0.897
Thighs not equal side to side during flight	U12 (*n* = 209)	111 (96.10%)	89 (95.20%)	0.758
	U14 (*n* = 153)	77 (93.50%)	70 (98.00%)	0.248
	U16 (*n* = 110)	43 (89.50%)	57 (92.20%)	0.640
	U17 (*n* = 103)	40 (83.80%)	50 (91.10%)	0.313
Foot placement not shoulder width apart	U12 (*n* = 209)	102 (88.20%)	87 (94.00%)	0.163
	U14 (*n* = 153)	75 (91.90%)	67 (94.10%)	0.653
	U16 (*n* = 110)	43 (89.50%)	47 (75.00%)	0.075
	U17 (*n* = 103)	45 (94.60%)	43 (77.80%)	0.032 *
Foot placement not parallel (front to back)	U12 (*n* = 209)	108 (92.90%)	87 (94.00%)	0.763
	U14 (*n* = 153)	77 (93.50%)	67 (94.10%)	0.901
	U16 (*n* = 110)	44 (92.10%)	52 (84.40%)	0.258
	U17 (*n* = 103)	44 (91.90%)	55 (100%)	0.052
Foot contact timing not equal (asymmetrical landing)	U12 (*n* = 209)	102 (88.20%)	84 (90.40%)	0.622
	U14 (*n* = 153)	75 (91.90%)	59 (82.40%)	0.124
	U16 (*n* = 110)	38 (78.90%)	50 (81.20%)	0.777
	U17 (*n* = 103)	39 (81.10%)	48 (86.70%)	0.491
Excessive landing contact noise	U12 (*n* = 209)	95 (81.90%)	82 (88.00%)	0.238
	U14 (*n* = 153)	73 (88.70%)	57 (80.40%)	0.218
	U16 (*n* = 110)	44 (92.10%)	50 (81.20%)	0.134
	U17 (*n* = 103)	39 (81.10%)	43 (77.80%)	0.713
Pause between jumps	U12 (*n* = 209)	98 (84.30%)	82 (88.00%)	0.454
	U14 (*n* = 153)	66 (80.60%)	61 (86.30%)	0.426
	U16 (*n* = 110)	38 (78.90%)	49 (79.70%)	0.929
	U17 (*n* = 103)	39 (81.10%)	39 (71.10%)	0.295
Technique declines prior 10 s	U12 (*n* = 209)	106 (91.30%)	86 (92.80%)	0.710
	U14 (*n* = 153)	77 (93.50%)	68 (96.10%)	0.551
	U16 (*n* = 110)	44 (92.10%)	57 (92.20%)	0.988
	U17 (*n* = 103)	44 (91.90%)	45 (82.20%)	0.201
Does not land in same footprint (consistent point of landing)	U12 (*n* = 209)	107 (92.10%)	86 (92.80%)	0.863
	U14 (*n* = 153)	73 (88.70%)	68 (96.10%)	0.150
	U16 (*n* = 110)	44 (92.10%)	58 (93.70%)	0.751
	U17 (*n* = 103)	40 (83.80%)	45 (82.20%)	0.852

Data are reported as *n* (%). *p* values were obtained by chi square test. * statistically significant (*p* < 0.05).

## Data Availability

The study did not report any data.

## References

[1] Drakos M.C., Domb B., Starkey C., Callahan L., Allen A.A. (2010). Injury in the National Basketball Association: A 17-year overview. Sports Health.

[2] Hootman J.M., Dick R., Agel J. (2007). Epidemiology of collegiate injuries for 15 sports: Summary and recommendations for injury prevention initiatives. J. Athl. Train..

[3] Rodas G., Bove T., Caparrós T., Langohr K., Medina D., Hamilton B., Sugimoto D., Casals M. (2019). Ankle Sprain Versus Muscle Strain Injury in Professional Men’ss Basketball: A 9-Year Prospective Follow-up Study. Orthop. J. Sports Med..

[4] Cherni Y., Jlid M.C., Mehrez H., Shephard R.J., Paillard T., Chelly M.S., Hermassi S. (2019). Eight Weeks of Plyometric Training Improves Ability to Change Direction and Dynamic Postural Control in Female Basketball Players. Front. Physiol..

[5] Taylor J.B., Wright A.A., Dischiavi S.L., Townsend M.A., Marmon A.R. (2017). Activity demands during multidirectional team sports: A systematic review. Sports Med..

[6] Andreoli C.V., Chiaramonti B.C., Buriel E., Pochini A.C., Ejnisman B., Cohen M. (2018). Epidemiology of sports injuries in basketball: Integrative systematic review. BMJ Open Sport Exerc. Med..

[7] Clifton D.R., Hertel J., Onate J.A., Currie D.W., Pierpoint L.A., Wasserman E.B., Knowles S.B., Dompier T.P., Comstock R.D., Marshall S.W. (2018). The first decade of web-based sports injury surveillance: Descriptive epidemiology of injuries in US High School Girls’ Basketball (2005–2006 through 2013–2014) and National Collegiate Athletic Association Women’s Basketball (2004–2005 through 2013–2014). J. Athl. Train..

[8] Clifton D.R., Onate J.A., Hertel J., Pierpoint L.A., Currie D.W., Wasserman E.B., Knowles S.B., Dompier T.P., Marshall S.W., Comstock R.D. (2018). The first decade of web-based sports injury surveillance: Descriptive epidemiology of injuries in US High School Boys’ Basketball (2005–2006 through 2013–2014) and National Collegiate Athletic Association Men’s Basketball (2004–2005 through 2013–2014). J. Athl. Train..

[9] Pasanen K., Ekola T., Vasankari T., Kannus P., Heinonen A., Kujala U.M., Parkkari J. (2017). High ankle injury rate in adolescent basketball: A 3-year prospective follow-up study. Scand. J. Med. Sci. Sports.

[10] Zuckerman S.L., Wegner A.M., Roos K.G., Djoko A., Dompier T.P., Kerr Z.Y. (2018). Injuries sustained in National Collegiate Athletic Association men’s and women’s basketball, 2009/2010–2014/2015. Br. J. Sports Med..

[11] Leppänen M., Pasanen K., Kannus P., Vasankari T., Kujala U.M., Heinonen A., Parkkari J. (2017). Epidemiology of overuse injuries in youth team sports: A 3-year prospective study. Int. J. Sports Med..

[12] Emery C.A. (2003). Risk factors for injury in child and adolescent sport: A systematic review of the literature. Clin. J. Sport Med..

[13] Agresta C., Church C., Henley J., Duer T., O’Brien K. (2017). Single-Leg Squat Performance in Active Adolescents Aged 8-17 Years. J. Strength Cond. Res..

[14] Ereña J.L. (2003). La planificación del entrenamiento como posible causa generadora de lesiones en el baloncesto. Proceedings of Jornadas Sobre Prevención de Lesiones en Baloncesto.

[15] Moseid C.H., Myklebust G., Slaastuen M.K., Bar-Yaacov J.B., Kristiansen A.H., Fagerland M.W., Bahr R. (2019). The association between physical fitness level and number and severity of injury and illness in youth elite athletes. Scand. J. Med. Sci. Sports.

[16] Monfort S.M., Comstock R.D., Collins C.L., Onate J.A., Best T.M., Chaudhari A.M. (2015). Association between ball-handling versus defending actions and acute noncontact lower extremity injuries in high school basketball and soccer. Am. J. Sports Med..

[17] Bahr R., Krosshaug T. (2005). Understanding injury mechanisms: A key component of preventing injuries in sport. Br. J. Sports Med..

[18] Cook G., Burton L., Hoogenboom B.J., Voight M. (2014). Functional movement screening: The use of fundamental movements as an assessment of function—part 1. Int. J. Sports Phys. Ther..

[19] Prangley I., Joyce D., Lewindon D. (2016). Assessing and developing the kinetic chain. Sports Injury Prevention and Rehabilitation: Integrating Medicine and Science for Performance Solutions.

[20] Steele J., Sheppard J., Joyce D., Lewindon D. (2016). Landing mechanics in injury prevention and performance rehabilitation. Sports Injury Prevention and Rehabilitation: Integrating Medicine and Science for Performance Solutions.

[21] Taylor J.B., Ford K.R., Hegedus E.J., Laver L., Kocaoglu B., Cole B., Arundale A., Bytomski J., Amendola A. (2020). Biomechanics of Lower Extremity Movements and Injury in Basketball. Basketball Sports Medicine and Science.

[22] Taylor J.B., Ford K.R., Hegedus E.J., Laver L., Kocaoglu B., Cole B., Arundale A., Bytomski J., Amendola A. (2020). Biomechanics of Upper Extremity Movements and Injury in Basketball. Basketball Sports Medicine and Science.

[23] Torres L., Cuzzolin F., Laver L., Kocaoglu B., Cole B., Arundale A., Bytomski J., Amendola A. (2020). Strength Training for Basketball: A methodological framework based on basketball and player’s needs. Basketball Sports Medicine and Science.

[24] Alderete J.L., Osma J.J. (1999). Baloncesto: Técnica de Entrenamiento y Formación de Equipos Base.

[25] Schelling X., Torres-Ronda L. (2016). An integrative approach to strength and neuromuscular power training for basketball. Strength Cond. J..

[26] Bond C., Dorman J., Odney T., Roggenbuck S., Young S., Munce T. (2019). Evaluation of the functional movement screen and a novel basketball mobility test as an injury prediction tool for collegiate basketball players. J. Strength Cond. Res..

[27] Calatayud J., Martin F., Gargallo P., García-Redondo J., Colado J.C., Marín P.J. (2015). The validity and reliability of a new instrumented device for measuring ankle dorsiflexion range of motion. Int. J. Sports Phys. Ther..

[28] Bennell K.L., Talbot R.C., Wajswelner H., Techovanich W., Kelly D.H., Hall A.J. (1998). Intra-rater and inter-rater reliability of a weight-bearing lunge measure of ankle dorsiflexion. Aust. J. Physiother..

[29] Trojian T.H., McKeag D.B. (2006). Single leg balance test to identify risk of ankle sprains. Br. J. Sports Med..

[30] Ugalde V., Brockman C., Bailowitz Z., Pollard C.D. (2015). Single leg squat test and its relationship to dynamic knee valgus and injury risk screening. Phys. Med. Rehabil..

[31] Crossley K.M., Zhang W.J., Schache A.G., Bryant A., Cowan S.M. (2011). Performance on the single-leg squat task indicates hip abductor muscle function. Am. J. Sports Med..

[32] Whatman C., Hume P., Hing W. (2013). The reliability and validity of physiotherapist visual rating of dynamic pelvis and knee alignment in young athletes. Phys. Ther. Sport.

[33] Ageberg E., Bennell K.L., Hunt M.A., Simic M., Roos E.M., Creaby M.W. (2010). Validity and inter-rater reliability of medio-lateral knee motion observed during a single-limb mini squat. BMC Musculoskelet. Disord..

[34] Cook G., Burton L., Hoogenboom B. (2006). Pre-participation screening: The use of fundamental movements as an assessment of function—part 1. N. Am. J. Sports Phys. Ther..

[35] Svilar L. (2019). Essentials of Physical Performance in Elite Basketball: Testing, Training, Load Monitoring, Periodization and Recovery.

[36] Fort-Vanmeerhaeghe A., Montalvo A.M., Lloyd R.S., Read P., Myer G.D. (2017). Intra- and Inter-Rater Reliability of the Modified Tuck Jump Assessment. J. Sports Sci. Med..

[37] Herrington L., Myer G.D., Munro A. (2013). Intra and inter-tester reliability of the tuck jump assessment. Phys. Ther. Sport..

[38] Smits-Engelsman B., Klerks M., Kirby A. (2011). Beighton score: A valid measure for generalized hypermobility in children. J. Pediatr..

[39] Harris A.J. (1958). Manuel D’application Des Tests de Latéralité.

[40] Hewett T.E., Myer G.D., Ford K.R. (2006). Anterior cruciate ligament injuries in female athletes: Part 1, mechanisms and risk factors. Am. J. Sports Med..

[41] Murphy D.F., Connolly D.A., Beynnon B.D. (2003). Risk factors for lower extremity injury: A review of the literature. Br. J. Sports Med..

[42] Gonzalo-Skok O., Serna J., Rhea M.R., Marín P.J. (2017). Age differences in measures of functional movement and performance in highly youth basketball players. Int. J. Sports Phys. Ther..

[43] Tao H., Husher A., Schneider Z., Strand S., Ness B. (2020). The relationship between single leg balance and isometric ankle and hip strength in a healthy population. Int. J. Sports Phys. Ther..

[44] Halabchi F., Angoorani H., Mirshahi M., Pourgharib Shahi M.H., Mansournia M.A. (2016). The Prevalence of Selected Intrinsic Risk Factors for Ankle Sprain among Elite Football and Basketball Players. Asian J. Sports Med..

[45] Paz G.A., Maia M., Farias D., Santana H., Miranda H., Lima V., Herrington L. (2016). Kinematic analysis of knee valgus during drop vertical jump and forward step-up in young basketball players. Int. J. Sports Phys. Ther..

[46] Steffen K., Nilstad A., Kristianslund E.K., Myklebust G., Bahr R., Krosshaug T. (2016). Association between Lower Extremity Muscle Strength and Noncontact ACL Injuries. Med. Sci. Sports Exerc..

[47] Powers C.M. (2010). The influence of abnormal hip mechanics on knee injury: A biomechanical perspective. J. Orthop. Sports Phys. Ther..

[48] Kulas A.S., Hortobágyi T., DeVita P. (2012). Trunk position modulates anterior cruciate ligament forces and strains during a single-leg squat. Clin. Biomech..

[49] Shultz S.J., Schmitz R.J., Benjaminse A., Collins M., Ford K., Kulas A.S. (2015). ACL Research Retreat VII: An Update on Anterior Cruciate Ligament Injury Risk Factor Identification, Screening, and Prevention. J. Athl. Train..

[50] Delahunt E., Remus A. (2019). Risk Factors for Lateral Ankle Sprains and Chronic Ankle Instability. J. Athl. Train..

[51] Biabanimoghadam M., Motealleh A., Cowan S.M. (2016). Core muscle recruitment pattern during voluntary heel raises is different between patients with patellofemoral pain and healthy individuals. Knee.

[52] Cowling E.J., Steele J.R. (2001). Is lower limb muscle synchrony during landing affected by gender? Implications for variations in ACL injury rates. J. Electromyogr. Kinesiol..

[53] Butler R.J., Crowell H.P., Davis I.M. (2003). Lower extremity stiffness: Implications for performance and injury. Clin. Biomech..

[54] Podraza J.T., White S.C. (2010). Effect of knee flexion angle on ground reaction forces, knee moments and muscle co-contraction during an impact-like deceleration landing: Implications for the non-contact mechanism of ACL injury. Knee.

[55] Pollard C.D., Sigward S.M., Ota S., Langford K., Powers C.M. (2006). The influence of in-season injury prevention training on lower-extremity kinematics during landing in female soccer players. Clin. J. Sport Med..

[56] Fort-Vanmeerhaeghe A., Gual G., Romero-Rodríguez D., Unnitha V. (2016). Lower Limb Neuromuscular Asymmetry in Volleyball and Basketball Players. J. Hum. Kinet..

